# Effect of the new video laryngeal mask airway SaCoVLM on airway management in lateral laparoscopic urological surgery: A single center randomized controlled trial

**DOI:** 10.1038/s41598-024-51856-4

**Published:** 2024-01-25

**Authors:** Yongtao Sun, Min Zhang, Xiaojun Gao, Zhongquan Gao, Ting Zou, Yongle Guo, Mengjie Liu, Lina Chen, Xiaoning Zhang, Yang Liu, Hai Feng, Yuelan Wang

**Affiliations:** 1 Department of Anesthesiology, The First Affiliated Hospital of Shandong First Medical University and Shandong Provincial Qianfoshan Hospital, Shandong Institute of Anesthesia and Respiratory Critical Medicine, Jinan, 250014 China; 2 Department of Anesthesiology, Shandong Provincial Hospital Affiliated to Shandong First Medical University (Shandong Provincial Hospital), Jinan, 250014 China; 3 Shandong First Medical University and Shandong Academy of Medical Sciences, Jinan, 250014 China; 4 Department of Nursing, The First Affiliated Hospital of Shandong First Medical University and Shandong Provincial Qianfoshan Hospital, Jinan, 250014 China; 5 Department of Anesthesiology, Shandong Public Health Clinical Center, Shandong University, Jinan, 250013 China

**Keywords:** Medical research, Risk factors, Urology

## Abstract

There are few pertinent studies about the application of laryngeal mask airways (LMAs) in lateral decubitus surgery. Therefore, the aim of our study was to evaluate the effects of lateral position and pneumoperitoneum on oropharyngeal leak pressure (OLP) and ventilation efficiency for the LMA SaCoVLM. Patients undergoing elective retroperitoneal laparoscopic urological surgery were randomized 1:1 to the Supreme group or SaCoVLM group. The primary outcome was the OLP with LMA insertion. The secondary outcomes were the first-attempt success rate, insertion time, adjustment times, gastric tube success rate, LMA alignment accuracy, LMA removal time, regurgitation or aspiration, LMA blood staining, and incidence of adverse events 24 h after surgery. We recruited 70 patients to complete the study. Regardless of lateral position and pneumoperitoneum, the OLP was greater in the SaCoVLM group (n = 35) than in the Supreme group (n = 35), with a median difference of 4–7 cmH_2_O. The first-attempt success rate of the SaCoVLM group was higher than that of the Supreme group (91.4% vs. 77.1%, risk ratio (RR): 1.19; 95% CI 0.96 to 1.46, *P* = 0.188). Thus, in the lateral position with pneumoperitoneum, although the new video LMA SaCoVLM has a higher OLP than the LMA Supreme, both devices provide sufficient ventilation efficiency.

It has been established that the LMA Supreme, which has a greater oropharyngeal leak pressure (OLP), is safe for use in the majority of laparoscopic procedures^1–3^. Modifications and improvements to the original first generation SADs yielded second-generation of two-channel models, including separate ventilation and gastric access tubes and features of anatomical curvature. However, the safety of second-generation LMA during laparoscopy for patients a high risk of reflux and emergency gastrointestinal surgery is still debatable and not generally acknowledged^4,5^.

In recent years, anesthesia technology has been developed in terms of precision, intelligence, information and visualization. Video LMA also represents a future development trend^6–8^. The new LMA SaCoVLM (Zhejiang UE Medical Corp, Hangzhou, China) combines the upper glottic airway device with video laryngoscope tracheal intubation and has the functions of a double-tube LMA and an intubating laryngeal mask airway (ILMA). LMA placement and tracheal intubation can be visualized, the LMA position can be continuously monitored during the operation and adverse events such as LMA displacement can be handled in a timely manner (Fig. 1)^9^. In previous applications and studies, we found that the LMA SaCoVLM had outstanding advantages in airway management^7,10^. Even when morbidly obese patients' awake airways were managed, we concluded that the LMA SaCoVLM is simple to use, well tolerated, and appropriate for awake orotracheal intubation in patients with known difficult airways^7^.

**Figure 1:**
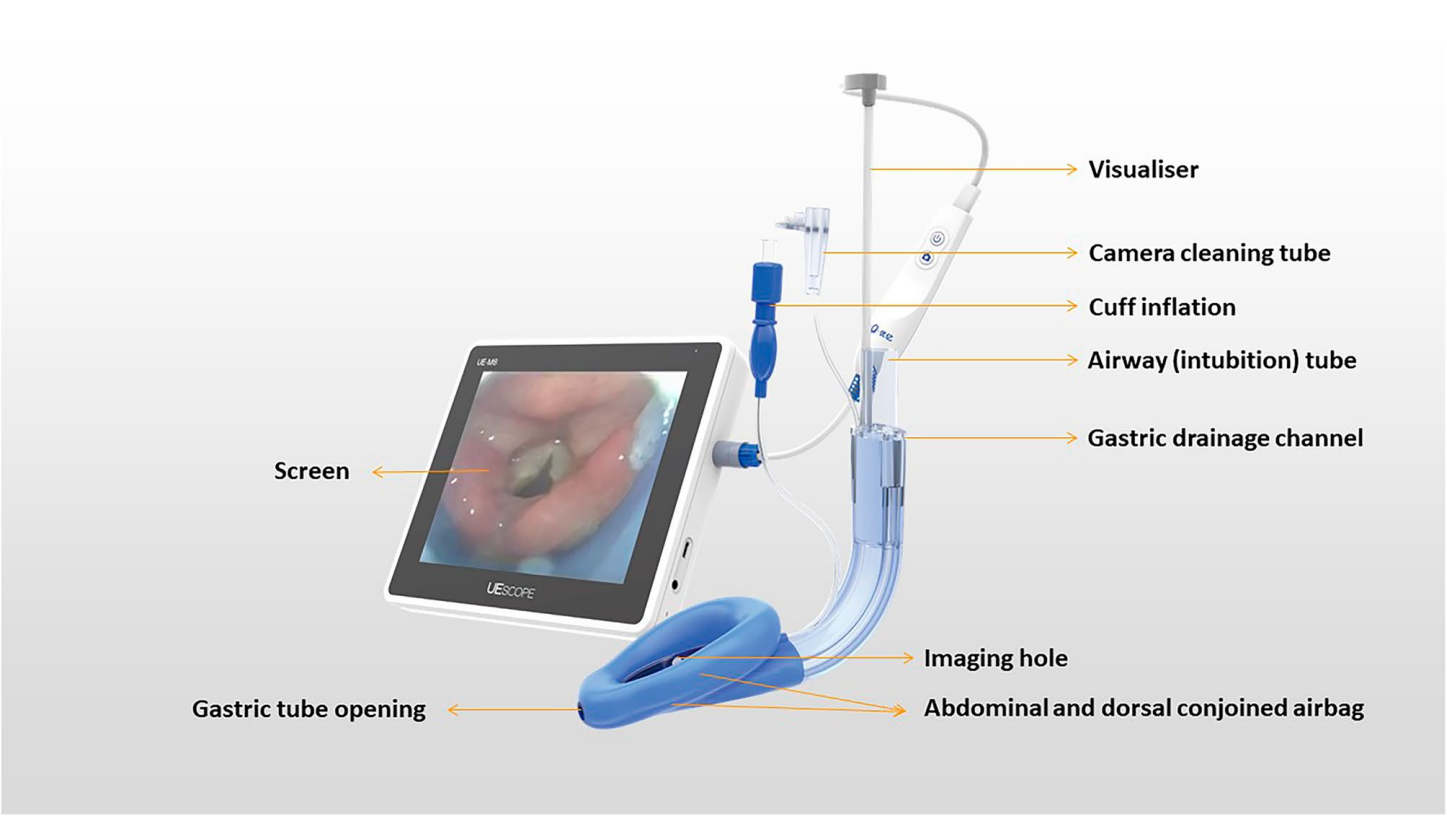
SaCoVLM (video intubation laryngeal mask airway). Photo courtesy of Zhejiang UE Medical Corp. (Hangzhou, China).

The LMA Supreme has been proven to be safe and effective for airway management in the lateral position and in laparoscopic surgery^2,3,10–12^, and the LMA SaCoVLM and Supreme have been used to the curvature of the oropharyngeal anatomical structure. Therefore, the LMA Supreme was selected as the control in this study. To our knowledge, this is the first study to investigate the role of video LMA in airway management under pneumoperitoneum in the lateral position. Therefore, in this study, we compared the effect of the lateral position and pneumoperitoneum on OLP as well as ventilatory efficiency with these two devices during laterally positioned laparoscopic surgery.

## Methods

This was a prospective, single-blind, parallel randomized controlled study. This study protocol was approved by the Shandong Provincial Qianfoshan Hospital Ethics Committee (YXLL-KY-2020 (046), 31/08/2020) and prospectively registered online at https://www.chictr.org.cn/index.aspx (registration identifier ChiCTR2000039502, 30/10/2020). All analyses and reports were completed in accordance with with the CONSORT reporting standard extension^13^. Informed consent was obtained from all participants and/or their legal guardians.

### Participants

Patients who underwent elective retroperitoneal laparoscopic urological surgery under general anesthesia were enrolled. The inclusion criteria for patients were as follows: American Society of Anesthesiologists (ASA) I- III; 18 ≤ age < 80 years; anticipated duration > 1 h; and 18 ≤ body mass index (BMI) < 35 kg m^-2^. The exclusion criteria for patients were as follows: a suspected or known difficult airway (Mallampati classification > III, interincisor distance < 2.5 cm, thyromental distance < 6 cm); severe gastrointestinal tract disease; patients undergoing emergency surgery and no fasting.

### Randomization and blinding

Following enrollment in the study, patients were randomized to either the Supreme group (n = 35) or SaCoVLM group (n = 35) by a computer-generated list. Sequentially numbered sealed opaque envelopes were kept by the research coordinator, and the investigators were blinded until 30 min before the induction of general anesthesia. The patients were not informed about the LMA used until the study was completed.

### Study design

**Figure 2:**
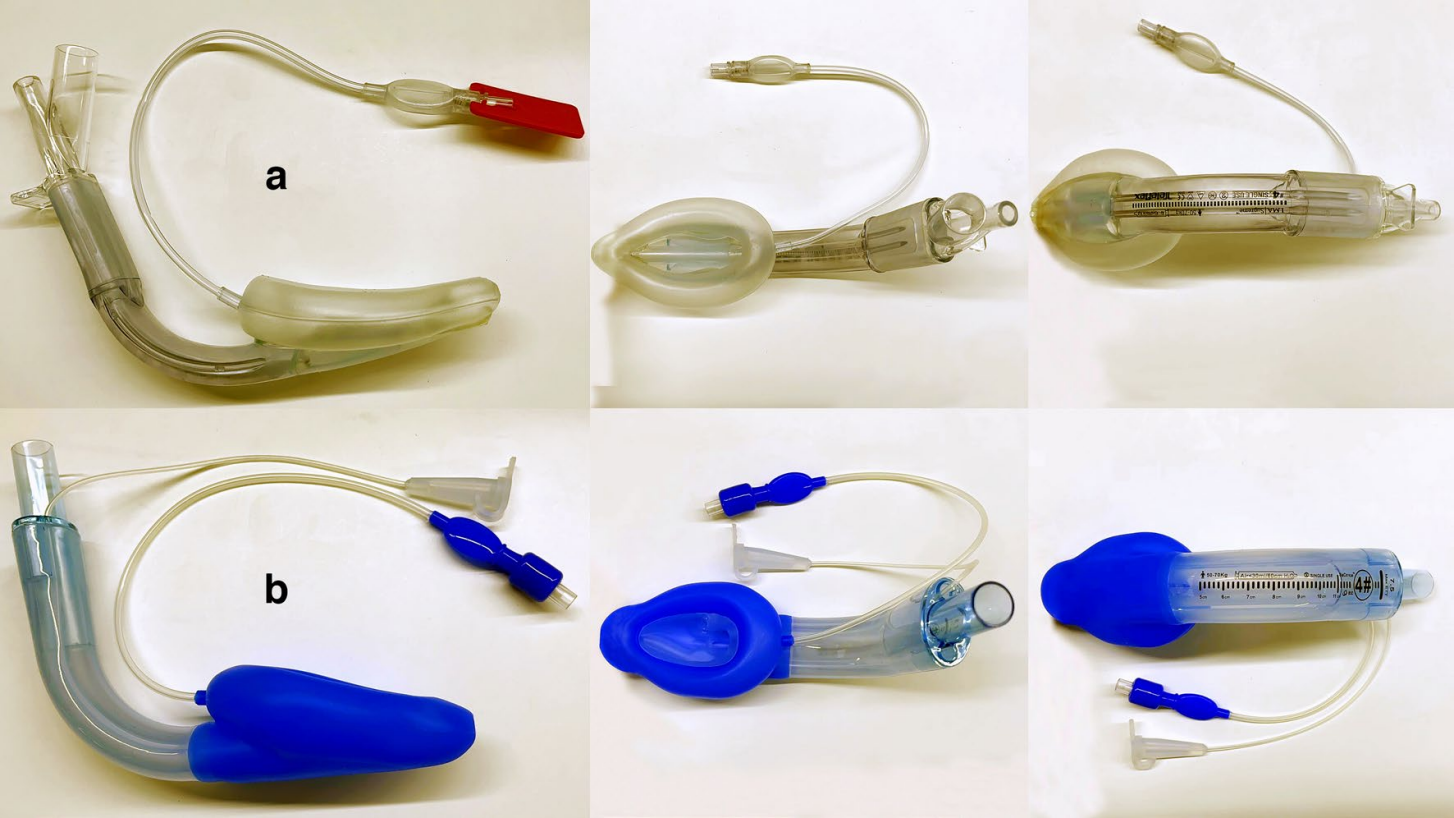
The LMA Supreme (**a**) and LMA SaCoVLM (**b**).

The sizes of the LMA Supreme and LMA SaCoVLM were chosen according to the manufacturer´s recommendations based on weight (size 3 for patients weighing 30 to 50 kg; size 4, 50 to 70 kg and size 5, > 70 kg). All LMAs were fully deflated and lubricated with a water-soluble gel, but using a different insertion technique. All insertions were performed by anesthetists who had extensive experience utilizing these SADs (≥ 100 LMA Supreme insertions; ≥ 50 LMA SaCoVLM insertions) (Fig. 2).

After standard fasting guidelines were followed, patients were taken to the operating room and standard American Society of Anesthesiologists (ASA) monitors (including continuous electrocardiography, noninvasive blood pressure, pulse oximetry, capnography, train-of-four stimulation and the bispectral index) were used. The patients did not receive any premedication. Anesthesia was induced with intravenous midazolam (0.04 mg kg^−1^), propofol (1.5–2.5 mg kg^−1^), and sufentanil (0.3–0.5 μg kg^−1^). No muscle relaxants were administered. When eyelash reflexes disappeared and BIS was less than 65, the patient's head was placed in sniffing the morning air position. The LMA was inserted after lubricating the cuff with a water based jelly. If resistance was encountered during insertion, the LMA was rotated. The cuff pressure was inflated to 60 cm H_2_O using a hand-held digital manometer (Covidien, Germany)^14,15^. Good bilateral chest undulation, the appearance of an end-tidal carbon dioxide (EtCO_2_) waveform and expiratory platform, minimal air leakage into the oropharynx, and a tidal volume of at least 6 mL kg^−1^ were needed for successful LMA placement. Depending on the situation, the LMA was adjusted by "up to down" or "lateral movements"^16^, or it was reinserted. If the number of LMA insertion attempts exceeded three, the insertion was considered to have failed. The patient was then intubated using a standard intubation technique and was eliminated from the trial.

Anesthesia was maintained with target-controlled infusions of propofol (2–4 μg ml^−1^) and remifentanil (3–5 ng ml^−1^). To maintain the neuromuscular blockade at one TOF twitch, further boluses of rocuronium (0.15 mg kg^−1^) were given. Patients were ventilated with intermittent positive pressure ventilation with a tidal volume of 6–8 mL kg^−1^, and I: E 1:2 and 10–12 breaths per minute were used for this study. The EtCO_2_ concentration was maintained between 35 and 45 mmHg, and the BIS was maintained between 40 and 60 during surgery. The pneumoperitoneal pressure was maintained between 10 and 12 mmHg for all procedures.

### Data collection

All the data were recorded by the same independent nonblind researcher. Our primary outcome was OLPs. The secondary outcomes were the first-attempt success rate, insertion time, number of LMA adjustments, success rate of gastric tube insertion, LMA alignment accuracy, LMA removal time, reflux or aspiration, LMA blood staining after removal, and incidence of complications within 24 h after surgery.

We measured the OLP at six time points (LMA insertion (T_0_), lateral position (T_1_), pneumoperitoneum (T_2_), 30 min after pneumoperitoneum (T_3_), 60 min after pneumoperitoneum (T_4_), and at the end of surgery (T_5_)). In manual ventilation mode, the adjustable pressure limitation was set to 40 cmH_2_O, and the oxygen flow rate was set to 3 L/min. When oropharyngeal air leakage occurred, the airway pressure was controlled by OLP. If there was no air leakage and the peak airway pressure (PAP) is greater than 40cmH_2_O, the test was stopped, and the OLP was noted as 40 cmH_2_O^12^. When measuring the OLP, the intracuff pressure was maintained at 40 cmH_2_O for all devices to eliminate the effect of intracuff pressure on the OLP^17^. We also recorded the peak airway pressure (PAP) at these time points. If the PAP exceeded the OLP and gas leakage was detected, we defined this as LMA leakage and recorded it^18^. Other intraoperative events such as hiccups, airway obstruction, reflux, or hypoxia were recorded.

**Figure 3:**
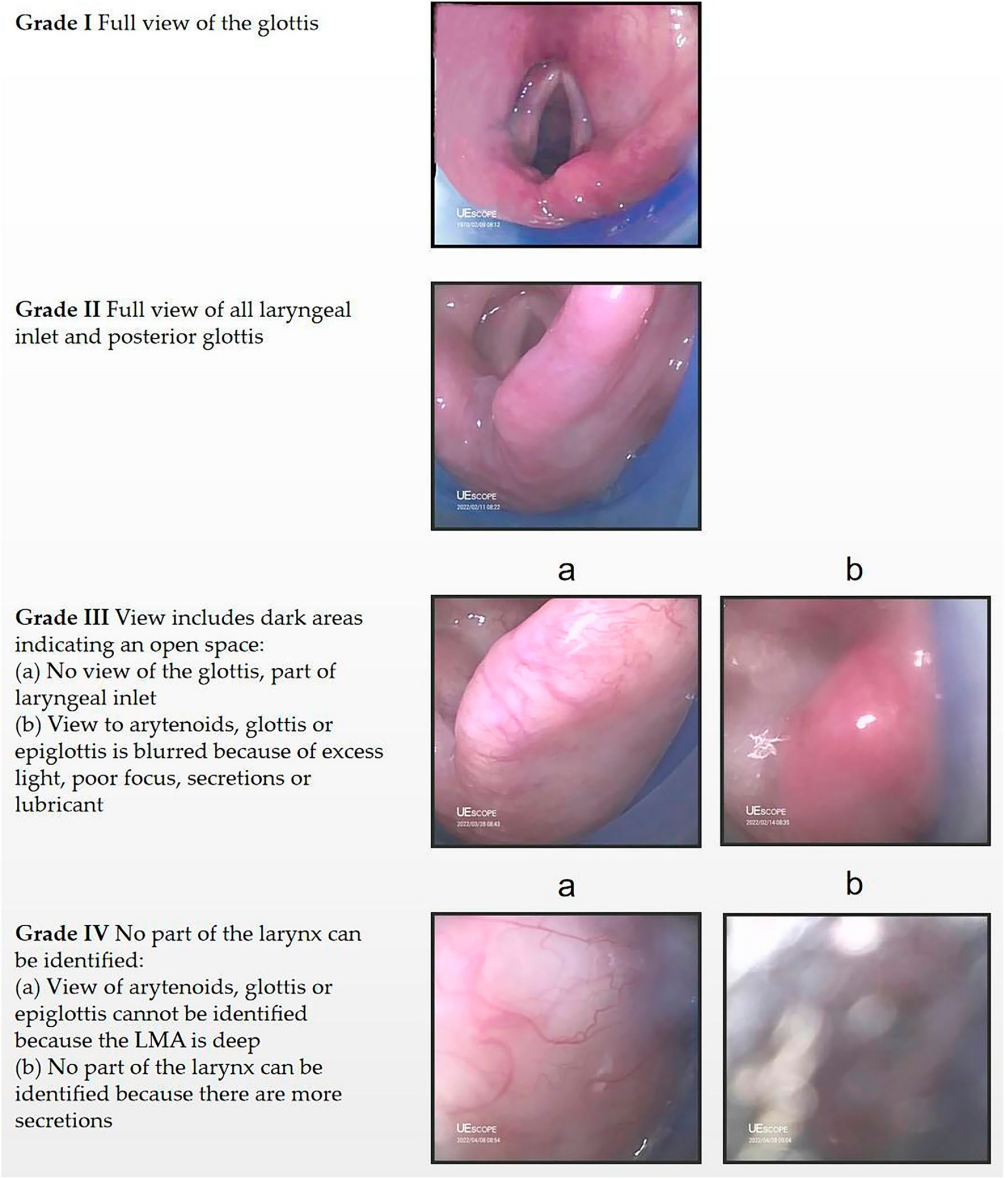
The LMA SaCoVLM glottic exposure grades. (Based on the suggestions of Timmermann A, Dhonneur G, and personal communication)^10,19,20^.

The insertion time was defined as the time from picking up the LMA to the appearance of three standard EtCO_2_ waveforms on the monitor. After the LMA was properly fixed, a well-lubricated 120 cm long #14 Salem sump gastric tube (Jinan Chensheng Medical Technology Co., Ltd., China) was inserted via the drain tube. The proportion of successful gastric tube insertions was recorded. After the OLP was measured, we used the Endoscopic View Grading System (EVGS) to evaluate the glottic imaging after successful LMA Supreme insertion and lateral positioning as follows: grade 1, the entire glottic aperture; grade 2, the local aperture of the glottis; grade 3, the free edge or tongue surface of the epiglottis; grade 4, no recognizable structure or sunscreen^9^. The LMA Supreme alignment accuracy was defined as an EVGS grade of 1 or 2. The LMA Supreme was introduced and advanced through a 3.8 mm fiberscope (LF-GP, Shirakawa, Olympus Co., Odakura, Nishigo-Mura, Japan) until the anterior medial edge of the cuff met the superior edge of the arytenoid cartilage. The LMA SaCoVLM grades the view of the laryngeal structure according to the criteria listed in Fig. 3. The LMA SaCoVLM alignment accuracy was defined as the view of a grade 1 or 2 laryngeal structure. The LMA SaCoVLM was inserted under visualization until the anterior medial edge of the cuff met the superior edge of the arytenoid cartilage.

The LMA removal time was defined as the time from stopping the anesthetic to removal. The reflux aspiration and LMA blood staining results after removal were recorded. Postoperative sore throat, dysphonia, and dysphagia within 24 h were monitored by a blinded independent observer. Sore throat was defined as persistent pain or discomfort in the throat that was unrelated to swallowing. Dysphonia was defined as difficulty speaking or pain while speaking. Dysphagia was defined as difficulty or pain caused by swallowing.

### Sample size

Based on previous studies^11^, the mean OLP expected for the LMA Supreme was 27 ± 4 cm H_2_O, and according to preliminary clinical data of the LMA SaCoVLM, the expected OLP was approximately 30 cm H_2_O. For a type I error of 0.05 and a power of 0.8, 28 patients were needed for each group. To cover a dropout rate of 20%, a total of 70 patients were included.

### Statistical analysis

For continuous variables, the normality of the data distribution was determined using the Shapiro‒Wilk test. The means ± SDs were used to represent normally distributed data, and two-sided Student's t tests were used to compare the data. Nonnormally distributed data are presented as the median (interquartile range, IQR) and were compared using the Mann‒Whitney U test. Categorical data are presented as percentage-based values and were compared with the χ^2^ test or Fisher’s exact test. The analysis of airway pressure and differences between OLP and airway pressure (normally distributed data) throughout the procedure were compared using repeated measures analysis of variance (ANOVA), and the analysis of OLP (nonnormally distributed data) throughout the procedure was compared using Friedman's two-way analysis. Bonferroni correction was used for multiple testing adjustments. We calculated the group differences or RRs with 95% confidence intervals (CIs), and the pseudomedian difference was calculated with the use of the Hodges–Lehmann estimate based on the Mann‒Whitney U test. The *P* value was two-sided, And *P* < 0.05 was considered to indicate statistical significance. The data were analyzed using the statistical software SPSS, version 23 (SPSS Inc., Chicago, Illinois, USA). Bonferroni correction was used for multiple testing adjustments.

### Ethical approval and consent to participate

This study was conducted in accordance with the Declaration of Helsinki, and was approved by the Institutional Review Board (or Ethics Committee) of Shandong Provincial Qianfoshan Hospital (identification number YXLL-KY-2020 (046) on 31 August 2020).

## Results

**Figure 4:**
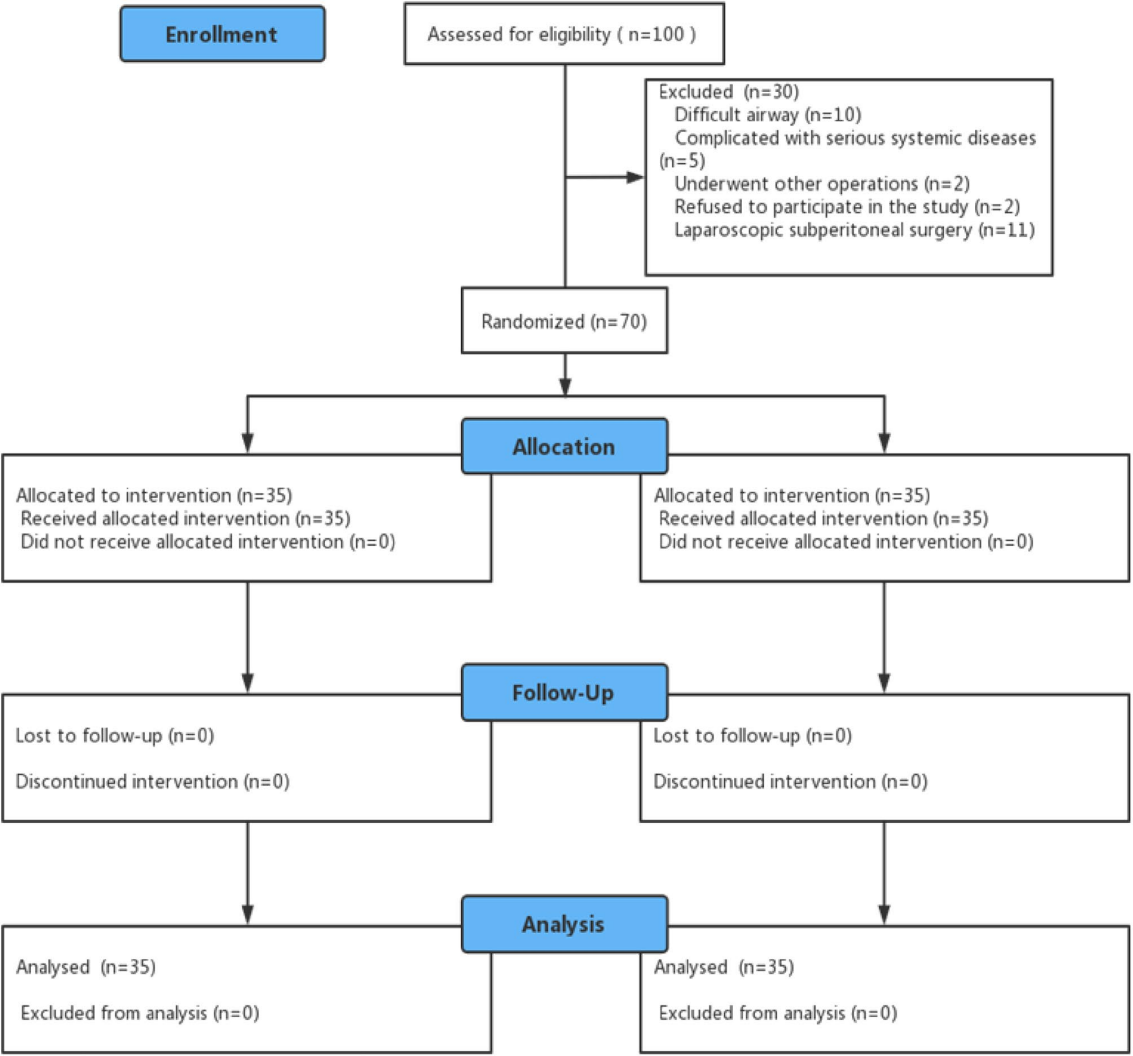
CONSORT flow diagram.

Of the 100 patients screened for eligibility, 30 were excluded, and 70 proceeded to randomization, with 35 patients randomized to the Supreme group and 35 randomized to the SaCoVLM group (Fig. 4). No significant differences were observed between the two groups in terms of baseline characteristics (Table 1).

**Table 1:** Patient characteristics and surgical data.

Patient characteristics	Supreme group (n = 35)	SaCoVLM group (n = 35)
Sex (male/female)	16/19	20/15
Age (years)	51.9 ± 11.1	50.4 ± 13.1
Weight (kg)	68.5 ± 11.6	70.9 ± 12.1
Height (cm)	164.8 ± 6.6	167.0 ± 8.0
BMI (kg/m^2^)	25.1 ± 3.4	25.3 ± 3.0
ASA (I/II/III)	1/32/2	0/35/0
Surgery position (left/right)	12/23	10/25
Mouth opening (cm)	4.5 (4 to 4.5)	4.3 (4.0 to 4.5)
Mallampati score (I/II/III)	6/22/7	5/25/5
Neck circumference (cm)	37.7 ± 3.8	38.9 ± 4.0
Thyromental distance (cm)	7.5 (7.0 to 8.0)	7.5 (6.5 to 8.0)
Surgical time (min)	164.0 ± 58.0	162.2 ± 47.2
Duration of anesthesia (min)	192.1 ± 53.9	192.6 ± 46.0
Type of surgery (n, %)		
Laparoscopic total nephrectomy	12 (34.3)	16 (45.7)
Laparoscopic partial nephrectomy	6 (17.1)	4 (11.4)
Laparoscopic adrenalectomy	11 (31.4)	12 (34.3)
Laparoscopic renal cyst decompression	5 (14.2)	2 (5.7)
Laparoscopic pyeloplasty	1 (2.9)	0 (0)
Laparoscopic perirenal and ureteral release	0 (0)	1 (2.9)

The values are presented as the mean ± SD, median (IQR) or number (proportion). BMI, body mass index; ASA, American Society of Anesthesiology.

### Primary outcome

**Figure 5:**
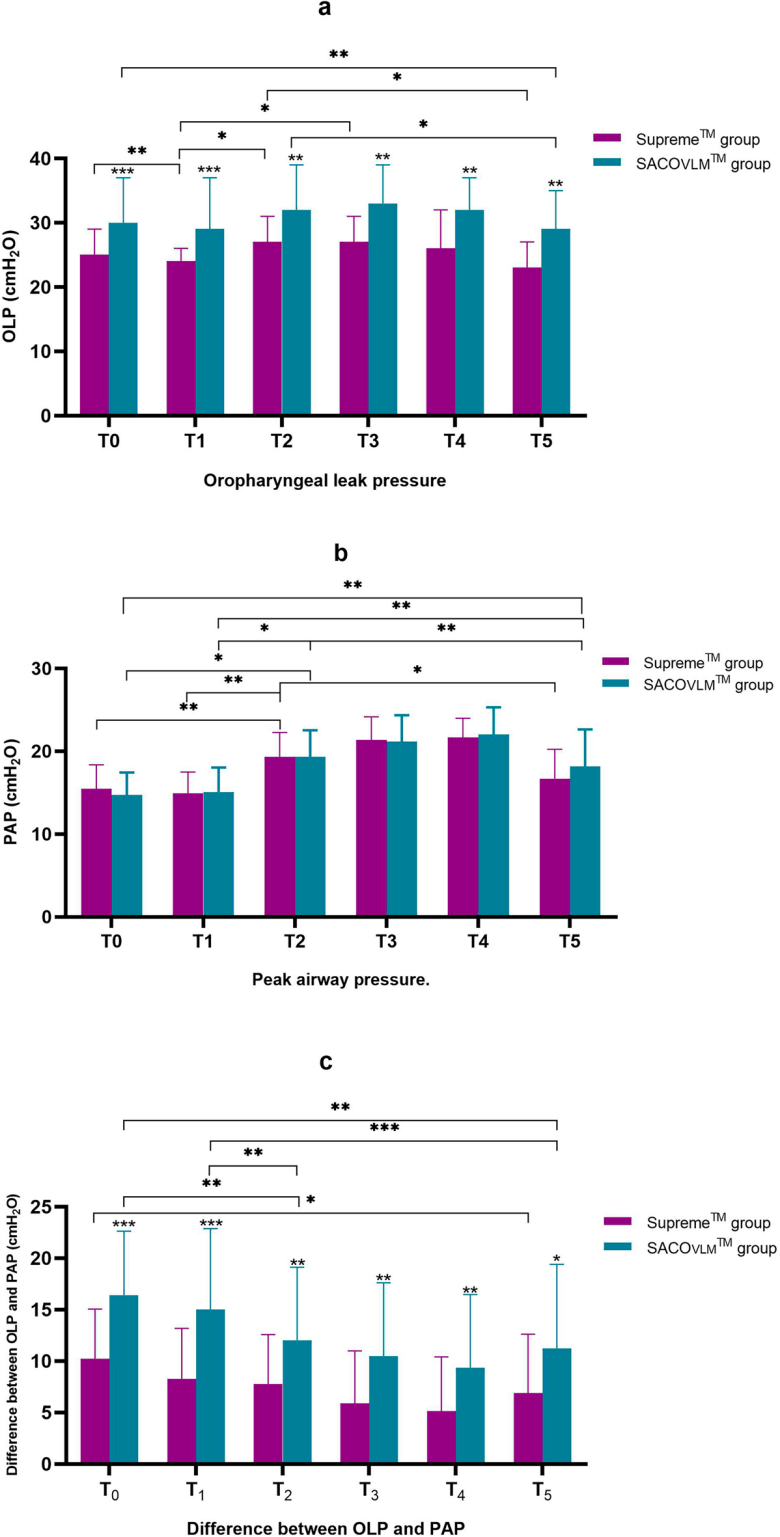
Oropharyngeal leak pressure and peak airway pressure at different times. T_0_, LMA insertion; T_1_, after lateral position; T_2_, after pneumoperitoneum; T_3_, 30 min after pneumoperitoneum; T_4_, 60 min after pneumoperitoneum; T_5_, surgery end. 95% CI, 95% confidence interval; OLP, oropharyngeal leak pressure; PAP, peak airway pressure.^*^P < 0.05, ^**^P < 0.01, ^***^P < 0.001.

In the supine position, the SaCoVLM group had a significantly greater OLP than did the Supreme group (30 (26 to 37) vs. 25 (22 to 29) cmH_2_O; group difference: 5; 95% CI: 3 to 8, *P* < 0.001) (Table 2). After lateral decubitus, the SaCoVLM group had a significantly greater OLP than did the Supreme group (29 (23 to 37) vs. 24 (20 to 26) cmH_2_O; group difference: 7; 95% CI: 4 to 10, *P* < 0.001), with a median difference of 5 cmH_2_O. At 30 min and 60 min after pneumoperitoneum and at the end of surgery, the SaCoVLM group had a significantly greater OLP. The median difference in the OLP between two groups was 5–6 cmH_2_O. Compared with that in the supine position, the OLP of the LMA Supreme was significantly lower in the lateral position (25 (22 to 29) vs. 24 (20 to 26) cmH_2_O; *P* = 0.007) but gradually increased after pneumoperitoneum and returned to the supine position. However, the OLP of the LMA SaCoVLM was unaffected by the lateral position or pneumoperitoneum (Fig. 5a).

**Table 2:** Oropharyngeal leak pressure and peak airway pressure at different times.

Time	Supreme group (n = 35)	SaCoVLM group (n = 35)	Group difference (95% CI)	*P* value
OLP (cmH_2_O)				
T_0_	25 (22 to 29)	30 (26 to 37)	5 (3 to 8)	< 0.001
T_1_	24 (20 to 26)*	29 (23 to 37)	7 (4 to 10)	< 0.001
T_2_	27 (23 to 31)^#^	32 (26 to 39)	4 (1 to 7)	0.008
T_3_	27 (23 to 31)^#^	33 (27 to 39)	4 (2 to 8)	0.006
T_4_	26 (23 to 32)^#^	32 (27 to 37)	5 (2 to 8)	0.002
T_5_	23 (20 to 27)^†^	29 (22 to 35) *^†^	6 (2 to 9)	0.001
PAP (cmH_2_O)				
T_0_	15.5 ± 2.9	14.7 ± 2.7	−0.74 (−2.09 to 0.60)	0.274
T_1_	14.9 ± 2.6	15.1 ± 2.9	0.17 (−1.15 to 1.49)	0.796
T_2_	19.3 ± 2.9*^#^	19.3 ± 3.2*^#^	0.00 (−1.46 to 1.46)	> 0.99
T_3_	21.4 ± 2.8 *^#^	21.2 ± 3.2*^#^	−0.23 (−1.65 to 1.20)	0.750
T_4_	21.7 ± 2.3*^#†^	22.0 ± 3.3*^#^	0.60 (−0.85 to 2.05)	0.621
T_5_	16.7 ± 3.5^†^	18.2 ± 4.5*^#†^	1.49 (−0.43 to 3.41)	0.127
Difference between OLP and PAP (cmH_2_O)				
T_0_	10.2 ± 4.8	16.4 ± 6.2	6.17 (3.51 to 8.83)	< 0.001
T_1_	8.3 ± 4.9	15.0 ± 7.9	6.74 (3.61 to 9.87)	< 0.001
T_2_	7.8 ± 4.8	12.0 ± 7.1*^#^	4.26 (1.36 to 7.16)	0.005
T_3_	5.9 ± 5.1*^#†^	10.5 ± 7.2*^#^	4.57 (1.61 to 7.53)	0.003
T_4_	5.1 ± 5.3 *^#†^	9.4 ± 7.1*^#†^	4.23 (1.24 to 7.21)	0.006
T_5_	6.9 ± 5.7*	11.2 ± 8.2*^#^	4.34 (0.97 to 7.72)	0.012

T_0_, LMA insertion; T_1_, after lateral position; T_2_, after pneumoperitoneum; T_3_, 30 min after pneumoperitoneum; T_4_, 60 min after pneumoperitoneum; T_5_, surgery end. 95% CI, 95% confidence interval; OLP, oropharyngeal leak pressure; PAP, peak airway pressure. Compared to T_0_, *P < 0.05; compared to T_1_, ^#^*p* < 0.05; compared to T_2_, ^†^*P* < 0.05.

The PAP increased significantly after pneumoperitoneum and gradually increased with prolongation of the pneumoperitoneum time. The mean OLP of the two groups was greater than the mean PAP (Table 2, Fig. 5b). The difference between OLP and PAP was significant at each time point (*P* < 0.05) (Table 2, Fig. 5c).

### Secondary outcomes

The LMA SaCoVLM had a longer insert time (70 (61 to 79) vs. 54 (49 to 65) seconds, difference: 13; 95% CI 7 to 19; *P* < 0.001). The number of patients requiring assisted rotational manipulation during insertion in the Supreme group was significantly lower than that in the SaCoVLM group (5 (14.3%) vs. 19 (54.3%) patients; RR, 3.8; 95% CI (1.6 to 9.0); *P* < 0.001) (Table 3).

**Table 3:** Details of airway management observed during surgery.

Management details	Supreme group (n = 35)	SaCoVLM™ group (n = 35)	Median difference or RR (95% CI)	*P* value
Insertion				
Number of attempts, n (%)				
1	27 (77.1)	32 (91.4)	1.19 (0.96 to 1.46)^a^	0.188
2	4 (11.4)	3 (8.5)	0.75 (0.18 to 3.11)^a^	> 0.99
3	4 (11.4)	0		0.114
Insertion time (s)	54 (49 to 65)	70 (61 to 79)	13 (7 to 19)	< 0.001
LMA rotation, n (%)	5 (14.3)	19 (54.3)	3.80 (1.60 to 9.04)^a^	< 0.001
Success rate of gastric tube placement, n (%)	35 (100)	35 (100)	NA	> 0.99
Glottic view				
Supine View grading (I/II/III/IV)	10/12/13/0	19/13/3/0		
LMA alignment accuracy in supine position, n (%)	22 (62.9)	32 (91.4)	1.455 (1.11 to 1.91)^a^	0.009
Lateral View grading (I/II/III/IV)	14/12/9/0	22/9/4/0		
LMA alignment accuracy in the lateral position, n (%)	26 (74.3)	31 (88.6)	1.192 (0.95 to 1.50)^a^	0.218
LMA removal time (s)	22 (13 to 31)	25 (16 to 38)	3 (-3 to 9)	0.318

The values are presented as the median (IQR), number (proportion), median difference (95% CI), or risk ratio (95% CI). CI, confidence interval; NA, not available; RR, risk ratio; ^a^risk ratio (95% CI).

In the supine position, the LMA SaCoVLM alignment accuracy was significantly greater than that of the Supreme (91.4% vs. 62.9, RR = 1.5; 95% CI = 1.1 to 1.9, *P* = 0.009). The EVGS grade in both the supine and lateral position groups was Grade I-III. The LMA Supreme EVGS grade was upgraded from Grade II to I in three patients and from Grade III to II in three patients and was Grade I in one patient, after the supine position was changed to the lateral position. The LMA Supreme alignment accuracy was increased to 74.3%. The LMA EVGS grade of SaCoVLM was upgraded from Grade II toI in six patients and from Grade I to II in one patient and was Grade III in one patient. The LMA alignment accuracy of SaCoVLM was reduced to 88.6%. (Table 3).

Between-group differences are expressed as pseudomedian differences calculated with the use of the Hodges–Lehmann estimate based on the Mann–Whitney U test. 95% CI, 95% confidence interval.

The incidence of intraoperative complications was lower in the two groups, and there was no reflux aspiration. However, the incidence of blood staining at the LMA SaCoVLM was higher than that at the LMA Supreme (22.9% vs. 2.9%, RR = 8.0; 95% CI = 1.1 to 60.6, *P* = 0.028). There was no significant difference in the incidence of complications within 24 h after the operation. (Table 4).

**Table 4:** Incidences of LMA insertion–related complications in the two groups, n (%).

	Supreme group (n = 35)	SACOVLM group (n = 35)	RR (95% CI)	*P* value
Leakage	7 (20)	3 (8.6)	0.43 (0.12 to 1.52)	0.306
Regurgitation (drain tube)	0 (0)	0 (0)	NA	> 0.99
Blood staining	1 (2.9)	8 (22.9)	8.0 (1.1 to 60.6)	0.028
Sore throat	5 (14.3)	5 (14.3)	NA	> 0.99
Hoarseness	9 (25.7)	9 (25.7)	NA	> 0.99
Dysphagia	4 (11.4)	2 (5.7)	0.5 (0.1 to 2.6)	0.673

Values are the number (proportion) or risk ratio (95% CI). CI, confidence interval; NA, not available.

## Discussion

In this prospective, single-blind, randomized controlled study of a new video LMA, both the LMA Supreme and the LMA SaCoVLM were successfully inserted, providing an effective airway with a low complication rate. These results are consistent with previous reports indicating the feasibility and effectiveness of LMA Supreme insertion in the lateral decubitus position during urological procedures^21^. However, we found slight but significant differences in the clinical performance of the two devices. Compared to the LMA Supreme, the LMA SaCoVLM also had a greater OLP not only in the supine position but also in the lateral position with or without pneumoperitoneum, and the difference between the values reached 4–7 cmH_2_O. It has been confirmed that SADs placed under direct vision are safer and more effective than SADs placed under direct vision^22^, indicating that the use of LMA SaCoVLM in the lateral position pneumoperitoneum is safer and that it can be used as an effective supraglottic airway management tool^8,9^.

When SADs are used, the OLP test is usually performed to quantify the seal with the airway. The OLP value has been widely used as a reference for evaluating the safety of different SADs. This score can indicate the success of positive pressure ventilation and the degree of airway protection^23^. When testing the suitability of LMA for laparoscopic surgery, the OLP is regarded as the most important parameter^3,24^.

The results showed that the mean OLP of both LMA groups was greater than the mean PAP, suggesting meaning that LMAs can provide effective ventilation for urologic laparoscopic surgery in the lateral position. Lan s et al.^13^ reported that the OLP of LMA Supreme was lower in the lateral position than in the supine position, and our results were consistent with these findings. However, our study showed that the OLP of LMA Supreme increased again in the lateral position after pneumoperitoneum. Another study involving 25 patients who underwent laparoscopic urological surgery revealed that the OLP of LMA Proseal increased significantly after pneumoperitoneum in the lateral position compared to that before pneumoperitoneum^25^. We also reached a similar conclusion. At this time, an increase in the OLP can just meet the needs of ventilation, which is the necessary function of LMA. In addition, our results showed that the difference between the OLP and PAP decreased gradually after use of the lateral position and pneumoperitoneum and tended to decrease gradually with time. A limitation is that we observed for only 60 min after pneumoperitoneum, and the evaluation time should be extended in the future studies. The difference between the OLP and PAP of LMA SaCoVLM was significantly greater than that of the LMA Supreme (9.4 ± 7.1 vs. 5.1 ± 5.3 cmH_2_O, *P* = 0.006). Neither the lateral position nor the pneumoperitoneum had a significant effect on the OLP of LMA SaCoVLM, possibly because the LMA SaCoVLM has an abdominal dorsal conjoined airbag with a wide and thick gourd-shaped cuff design on the front. Increasing the bonding area with oropharyngeal tissue is one of the important factors affecting OLP^8,9,26^, which suggests that the LMA SaCoVLM can be safely and effectively used in lateral laparoscopic surgery. In addition, when the OLP decreased and the PAP increased, both LMA groups were able to maintain normal EtCO_2_ levels by adjusting the minute ventilation in most patients.

Ten patients experienced LMA leakage in the two groups during the operation, with such leakage occurring 30 min after pneumoperitoneum in six patients. LMA leakage after pneumoperitoneum is one of the concerns of anesthesiologists when using LMAs^2,3,27^. Although 10 patients experienced leakage during the LMA operation, eight patients did not experience a change in the tidal volume and were not given special treatment. Only two patients with the LMA Supreme needed to have their ventilator parameters adjusted or their dosage of muscle relaxant increased to eliminate the obvious leakage sound heard at the LMA, which shows that most LMA leakage has no clinical significance.

Another important aspect that should be considered is the maneuverability of LMA. The insertion time of the LMA SaCoVLM was longer (70[61 to 79] s vs. 54 [49 to 65] s). We analyzed the reason for the differences in the materials used between the two devices. The cover body and handle of the LMA SaCoVLM are made of silicone, which has a soft texture. A wide and thick gourd-shaped mask bag at the front increases the length of the LMA. In the actual operation process, it is usually necessary to insert the endoscope laterally using the rotation technique, which prolongs the insertion time. A more effective insertion method needs to be identified. The cover handle of the LMA Supreme is made of PVC, which is a harder material and makes it easier to control the insertion direction. The first-attempt success rate of LMA SaCoVLM was 91.4%, which was greater than that of LMA Supreme (77.1%), but no significant differences were observed between them. Yan et al.^28^ reported100 adult patients who were treated with LMA SaCoVLM for general anesthesia. The first-attempt success rate was 95%. Li et al.^29^ studied 408 adult patients to determine whether a new LMA Supreme insertion technique (not removing the pilot tube blocker before insertion) lowers the incidence of sore throat in the postanesthesia care unit (PACU). Their data showed that the first-attempt success rates of the two groups were 73.5% and 85.3%, respectively, and the overall success rate was 100%. We obtained similar research results.

Possible LMA displacement after the lateral position is an important factor that affects the choice of airway management tools by the anesthesiologist. To compare the effects of the two LMA placements, we used the EVGS grade to evaluate the exposure of the pharyngeal anatomy. Furthermore, we also designed the glottic exposure classification standard under the LMA SaCoVLM with reference to the EVGS classification and previous studies^10,19,20^, hoping to provide guidance for the development of video LMA technology. The data obtained in this study showed that the LMA alignment accuracy of SaCoVLM was significantly greater than that of Supreme in the supine position (91.4% vs. 62.9%). After the supine position was changed to the lateral position, the LMA Supreme alignment accuracy was improved to 74.3%. The LMA SaCoVLM alignment accuracy decreased to 88.6%, and the LMA alignment accuracy in the lateral position was similar between the two groups, which was similar to the results of previous studies^2,17,30,31^. Our research results provide a good theoretical basis for the safety of LMA in lateral-position surgery from the perspective of fiber optics.

Low complication rates were recorded for both devices during the maintenance of anesthesia, with air leakage rates of 8.6% for the LMA SaCoVLM and 20% for the LMA Supreme. No significant differences were observed between the two groups, and no reflux aspiration occurred, which was similar to the results of previous studies^10,30^. In terms of the incidence of postoperative complications, the incidence of blood staining in the SaCoVLM group was higher than that of the Supreme group (22.9% vs. 2.9%), which is quite different from the 7% incidence of blood staining reported by Yan et al.^28^, additional research is needed for confirmation. No significant differences were observed in the incidence of complications within 24 h after the operation, and no severe pharyngeal pain, hoarseness, or dysphagia was found; moreover, these symptoms were all mild and relieved within 24 h after the operation^10,11,30,32^.

Nonetheless, our study has several limitations. First, although the postoperative observer and patients were blinded to the group distribution, the anesthesiologist was not blinded to the type of LMA used, which might result in bias. Second, OLP data may not be appropriate for patients with difficult airways because this was the exclusion criterion in our study. Third, the LMA SaCoVLM camera is designed to be on the right side, not in the center. The glottis cannot be completely imaged technically on the screen, affecting the image classification. Finally, in this study, patients were ventilated with tidal volume of 6–8 mL kg^−1^, which might have affected peak airway pressure being measured.

## Conclusions

In the lateral position and under pneumoperitoneum, the LMA SaCoVLM and Supreme both provided considerable ventilation efficiency. Our data showed that the new video LMA SaCoVLM has a greater OLP. Moreover, the first-attempt success rate and LMA alignment accuracy of SaCoVLM were higher. Despite the longer insertion time and the greater incidence of blood staining in the SaCoVLM group, it was proven that the application of visualization technology can effectively improve the safety of LMA airway management. Visualization and a higher OLP can allow SGAs to be used in wider ranges of patients and procedures.

## Data Availability

The datasets used and analyzed during this study are available from the corresponding author upon reasonable request.

## Abbreviations

OLP: Oropharyngeal leak pressure
LMA: Laryngeal mask airway
RR: Risk ratio
SAD: Supraglottic airway device
ILMA: Intubating laryngeal mask airway
ASA: American Society of Anesthesiologists
BMI: Body mass index
TOF: Train-of-four
BIS: Bispectral index
TCI: Target-controlled infusion
EVGS: Endoscopic view grading system
ANOVA: Analysis of variance
CI: Confidence interval
