# Author Correction: Effect of the new video laryngeal mask airway SaCoVLM on airway management in lateral laparoscopic urological surgery: a single center randomized controlled trial

**DOI:** 10.1038/s41598-024-54129-2

**Published:** 2024-02-15

**Authors:** Yongtao Sun, Min Zhang, Xiaojun Gao, Zhongquan Gao, Ting Zou, Yongle Guo, Mengjie Liu, Lina Chen, Xiaoning Zhang, Yang Liu, Hai Feng, Yuelan Wang

**Affiliations:** 1 Department of Anesthesiology, Shandong Institute of Anesthesia and Respiratory Critical Medicine, The First Affiliated Hospital of Shandong First Medical University and Shandong Provincial Qianfoshan Hospital, Jinan, 250014 China; 2 Department of Anesthesiology, Shandong Provincial Hospital Affiliated to Shandong First Medical University (Shandong Provincial Hospital), Jinan, 250014 China; 3 Department of Nursing, The First Affiliated Hospital of Shandong First Medical University and Shandong Provincial Qianfoshan Hospital, Jinan, 250014 China; 4 Department of Anesthesiology, Shandong First Medical University, Jinan, 250014 China; 5 Department of Anesthesiology, Shandong Public Health Clinical Center, Jinan, 250013 China

Correction to: *Scientific Reports* 10.1038/s41598-024-51856-4, published online 25 January 2024

The original version of this Article contained an error in Affiliation 2, which was incorrectly duplicated and given as ‘Department of Anesthesiology, Shandong Public Health Clinical Center, Jinan 250013, China’. The correct affiliation is listed below:

Department of Anesthesiology, Shandong Provincial Hospital Affiliated to Shandong First Medical University (Shandong Provincial Hospital), Jinan 250014, China.

The original Article has been corrected.

